# Norwegian Scabies

**DOI:** 10.5811/westjem.2015.5.27383

**Published:** 2015-07-10

**Authors:** Patrick Burns, Shelley Yang, Jared Strote

**Affiliations:** * University of Washington, Department of Emergency Medicine, Seattle, Washington; † University of Washington, Department of Dermatology, Seattle, Washington

## PATIENT PRESENTATION

A 48-year-old male presented with body aches and a chronic rash. He had no medical history aside from two unsuccessful treatments for presumed scabies and a recent diagnosis of psoriasis. Physical exam revealed hypotension, tachycardia, and profound, diffuse yellow crusting of the skin with erythematous erosions covering non-crusted areas (Figure). The patient was resuscitated and treated for septic shock while microscopic evaluation of scrapings of the crusted skin was performed (Video).

## DIAGNOSIS

The patient had crusted (Norwegian) scabies with associated Enterobacter sepsis.

Crusted scabies is a rare skin infestation caused by *Scarcoptes scabiei* with parasitic loads in the thousands to millions.

In contrast to common scabies infections, it tends to affect immunosuppressed or debilitated patients and pruritus is not prominent. Patients present with scaly, hyperkeratotic, gray to erythematous plaques. Given its similarity to other dermatologic processes, misdiagnosis is common. Clinical diagnosis is aided by microscopic identification of scabies mites, eggs or feces in skin scrapings or under fingernails. Occasionally videodermatoscopy or biopsy is necessary. Skin breakdown can lead to cellulitis and systemic bacterial infection.^1^–^2^

Treatment for mild cases is the same as for uncomplicated scabies infections. For severe cases, oral or intravenous (IV) ivermectin should be given. 1

After resuscitation, the patient was admitted with broad-spectrum IV antibiotics and ivermectin as well as topical permethrin. A Human Immunodeficiency Virus test sent from the emergency department came back positive. After a prolonged hospital course with multiple complications from his skin breakdown and sepsis, he was discharged with full eradication of his scabies infestation.

## Figures and Tables

**Figure:**
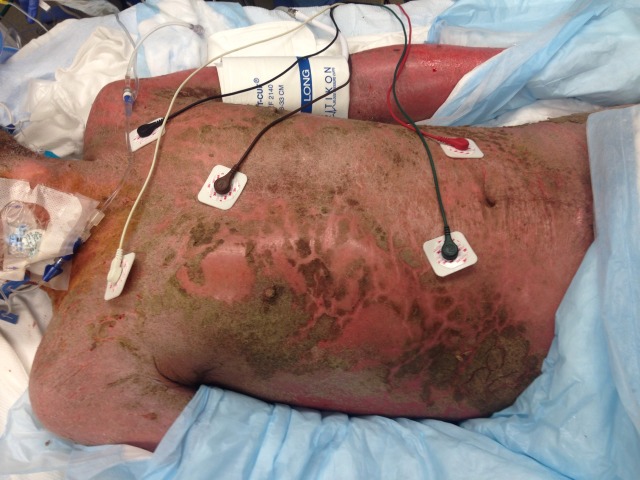
Photograph of the patient’s skin exam demonstrating diffuse crusting lesions and erythema.

**Video:** Videodermatoscopy of moving scabies mite.
